# Guided Wave-Convolutional Neural Network Based Fatigue Crack Diagnosis of Aircraft Structures †

**DOI:** 10.3390/s19163567

**Published:** 2019-08-15

**Authors:** Liang Xu, Shenfang Yuan, Jian Chen, Yuanqiang Ren

**Affiliations:** Research Center of Structural Health Monitoring and Prognosis, State Key Lab of Mechanics and Control of Mechanical Structures, Nanjing University of Aeronautics and Astronautics, Nanjing 210016, China

**Keywords:** convolutional neural network, guided wave based monitoring, fatigue crack diagnosis, uncertainty, structural health monitoring

## Abstract

Fatigue crack diagnosis (FCD) is of great significance for ensuring safe operation, prolonging service time and reducing maintenance cost in aircrafts and many other safety-critical systems. As a promising method, the guided wave (GW)-based structural health monitoring method has been widely investigated for FCD. However, reliable FCD still meets challenges, because uncertainties in real engineering applications usually cause serious change both to the crack propagation itself and GW monitoring signals. As one of deep learning methods, convolutional neural network (CNN) owns the ability of fusing a large amount of data, extracting high-level feature expressions related to classification, which provides a potential new technology to be applied in the GW-structural health monitoring method for crack evaluation. To address the influence of dispersion on reliable FCD, in this paper, a GW-CNN based FCD method is proposed. In this method, multiple damage indexes (DIs) from multiple GW exciting-acquisition channels are extracted. A CNN is designed and trained to further extract high-level features from the multiple DIs and implement feature fusion for crack evaluation. Fatigue tests on a typical kind of aircraft structure are performed to validate the proposed method. The results show that the proposed method can effectively reduce the influence of uncertainties on FCD, which is promising for real engineering applications.

## 1. Introduction

Damage monitoring is a key issue for safety-critical systems such as aircraft, wind turbines, bridges, and nuclear plants [1,2]. As a common damage type, fatigue crack is one of the primary causes of structural failure, which is estimated to cause up to 90% of failures of metallic structures in service [3]. It is of considerable significance to monitor fatigue crack state to ensure the safety of structures in engineering applications.

The guided wave (GW) based structural health monitoring method [4,5] has been widely investigated to realize fatigue crack diagnosis (FCD) [6]. However, reliable FCD in real engineering applications still meets challenges because of uncertainties in real engineering applications, such as crystal structures in material, performances of sensors, bonding between sensors and structures, propagation paths, or internal inclination angle of fatigue cracks, etc. These uncertainties usually cause serious change both to the crack propagation itself and GW monitoring signals. Due to these uncertainties, FCD calibration by one or even a set of training structures usually does not work well for a monitoring structure in service.

Some developments have been reported on dealing with the influence of uncertainties on damage diagnosis. The method of extracting GW damage features that are sensitive to damage changes instead of external factors (such as environmental temperature, surface humidity) has been investigated [7,8,9]. However, the performance of these GW damage features has only been verified in simple structures such as aluminum plates. In real engineering applications, uncertainties will make the baseline signals dispersive, thus reducing the reliability of damage diagnosis. Furthermore, machine learning techniques are useful in modeling complex nonlinear phenomena in the presence of uncertainties [10], which can be introduced to construct a mathematical model of damage features to estimate the damage of structures. Yuan and Mei et al. [11] proposed the GW-Hidden Markov model based method to achieve a probabilistic evaluation of the propagation state of cracks. Uncertainties such as changing load and structural boundary conditions were considered in this research. Qiu et al. [12,13] proposed the GW-Gaussian mixture model based method to model the probability characteristic of GW features under changing loads. In the above methods, the number of Gaussian components, parameter initialization, and convergence of the model are still problems that need to be further researched. Moreover, the artificial neural network is an effective mathematical method that is widely used for damage estimating, which can learn the relevant features from data. Gu et al. [14] utilized an artificial neural network to study the change of natural frequency of structure caused by damages at different temperatures. It is shown that the proposed artificial neural network with novelty detection is able to distinguish damage occurrence and severity regardless of temperature variations and noise perturbations. However, the verification is only conducted based on a simple supported beam and finite element models. Other methods, such as support vector machine [15], fuzzy logic [16], and so on have also made some progress. However, these methods still have drawbacks, such as limited hierarchy, and the optimization process is prone to fall into the local optimum leading to a limited expression and limited generalization ability to data. These methods still face restrictions in engineering applications.

In recent years, deep learning has attracted significant attention [17]. As one of the deep learning methods, the convolutional neural network (CNN) has been successfully applied in many areas [18,19]. CNN has a deep hierarchy and complex structure, which makes it more capable of expressing data. Its unique structures, such as local connection, shared weights, and sub-sampling, allow it to automatically extract representations that are beneficial for classification by fusing a large amount of data [20]. These characteristics make CNN owns the potential to be used to improve the diagnosis accuracy of crack length under the influence of uncertainties. However, research on damage diagnosis of mechanical structures using CNN is rarely reported. Sun et al. [21] put forward a discriminant convolutional feature learning method. In this method, the CNN model is utilized to derive invariant and robust features, and vibration signals are used to monitor the fault conditions of the induction motor. Six different fault conditions are identified, but the severity of fault conditions is not distinguished. Guo et al. [22] proposed a hierarchical learning rate adaptive CNN based on an improved algorithm, in which the CNN model is based on the classical LeNet5 models proposed by LeCun. This method achieved both fault-pattern recognition and fault-size evaluation. Although showing potential in addressing the uncertainty issue, the current CNN based damage diagnosis are mainly performed based on vibration-based methods, which are not sensitive to small damages like crack. Until now, there has been little research on combining and taking advantages of the GW based structural health monitoring and CNN, which could be used for reliable FCD under the influence of uncertainties and is urgently needed.

In this paper, a GW-CNN based FCD method is proposed to address the influence of uncertainties on reliable FCD. In this method, different kinds of damage indexes (DIs) from multiple GW exciting-acquisition channels under one crack length were extracted to construct the input feature vector. Then, a designed CNN model was trained with feature vectors at different crack lengths extracted from historical GW data. Finally, the feature vector obtained from the monitored structure forward propagates through the trained CNN layer by layer. High-level features are automatically extracted, which are beneficial for FCD. Reliable crack size can be directly obtained by the CNN output under the influence of uncertainties among different structures.

The rest of the article is organized as follows: in Section 2, details of the proposed method are shown. In Section 3, the feasibility of the proposed method is verified by attachment lug specimens, and the advantages are verified by comparative experiments. The conclusion is drawn in Section 4.

## 2. GW-CNN Based Fatigue Crack Diagnosis Method

In this section, the proposed GW-CNN based FCD method is introduced. Firstly, GW features that are extracted from multiple GW exciting-acquisition channels are used to construct the CNN input vector. Then, a CNN model is designed and trained for FCD. Finally, the trained CNN model can be used for reliable FCD of a monitored structure.

### 2.1. Multi-Channel and Multi-GW Features Extraction

**Figure 1:**
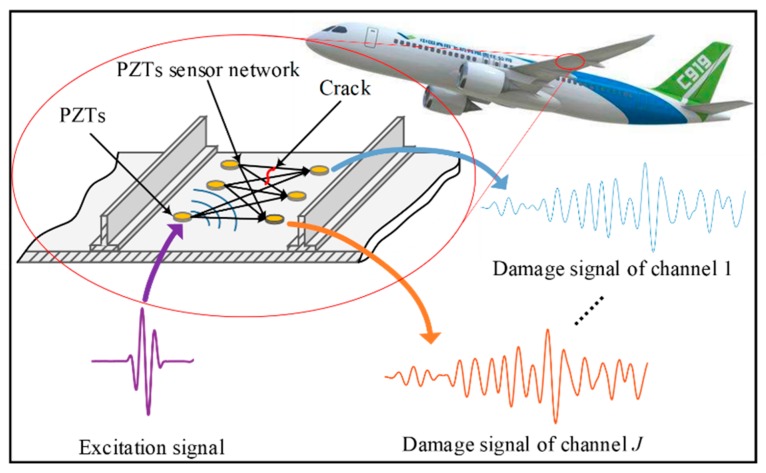
The guided wave (GW) based structural health monitoring method for on-line crack monitoring. PZT: Piezoelectric transducer.

GW is a kind of elastic waves that propagates in plate-like structures. It can travel a long distance in structures with small energy loss; hence, it can be used to monitor a relatively large structure area. Piezoelectric transducer (PZT) is a conventional kind of sensor used to excite and receive GW in structure. A typical sensor network configuration is shown in Figure 1 where *J* ($J\in N^{+}$) exciting-acquisition channels are formed. From one sensor network, the number of obtained GW signals is *J*. After the GW is excited in the structure by a PZT, the interaction of the GW with the crack can influence the GW propagation. By comparing the received signals under healthy and cracked conditions, the length of the crack can be estimated. 

Many kinds of DIs can be defined to evaluate the variations between the baseline signal collected when the structure is healthy and the monitoring signal when the crack propagates in the structure. These DIs are extracted from GW signals in the time domain, frequency domain, and time*–*frequency domain.

As mentioned above, various GW features can be obtained from one crack length, which provides the potential of reliable FCD through data fusion of CNN. Based on this, a multi-channel and multi-GW features extraction method is proposed as follows. Firstly, under the one crack length, GW signals from *J* exciting-acquisition channels are acquired, recorded as $S=\{s^{1},s^{2},\ldots ,s^{i},\ldots ,s^{J}\}$. Then, from one GW signal **s***^i^*, *d* kinds of DIs were chosen for GW features extraction, which were recorded as $D^{j}=[D_{1}^{j},D_{2}^{j},\ldots ,D_{d}^{j}]$, *j* ∈ {1,2,…,*J*}. Finally, the extracted DIs were arranged into a feature vector of **p** in order of channel as shown in Equation (1):

(1) $$p={\left[D_{1}^{1},D_{2}^{1},\ldots ,D_{1}^{j},D_{2}^{j},\ldots ,D_{d}^{j},\ldots ,D_{d-1}^{J},D_{d}^{J}\right]}_{(J\times d)\times 1}$$

The feature vector **p** was standardized to $p^{norm}$ to obtain a similar data distribution [23] for each input feature to achieve a better learning efficiency and improve the accuracy of FCD.

### 2.2. CNN Based Fatigue Crack Diagnosis

Based on the above procedure of data preprocessing, DIs from multi-channels are formed into a one-dimensional (1D) feature vector $p^{norm}$, which is designed as the CNN input. Crack lengths are divided into *z* ($z\in N^{+}$) crack sizes for classification; each crack size is an interval that contains a certain crack length. Softmax classifier [24] is adopted as the output layer to express *z* crack sizes, which makes the CNN output a vector that has the form of $a={\left[a_{1},a_{2},\ldots ,a_{k},\ldots ,a_{z}\right]}_{z\times 1}$. The classification result corresponds to the class that has the highest output value. For example, the desired output **q**, which denotes the *k*th crack size, is defined as a vector with *z* elements as $q={\left[q_{1},q_{2},\ldots ,q_{k},\ldots ,q_{z}\right]}_{z\times 1}$, in which the *k*th element value *q_k_* = 1 and the rest are 0. 

In this study, the GW signals corresponding to crack lengths are obtained from known structures, which are called historical data. The feature vector $p^{norm}$ obtained from historical data and its corresponding desired output **q** is formed into an input sample {$p^{norm},q$}. These historical input samples are utilized for CNN training.

**Figure 2:**
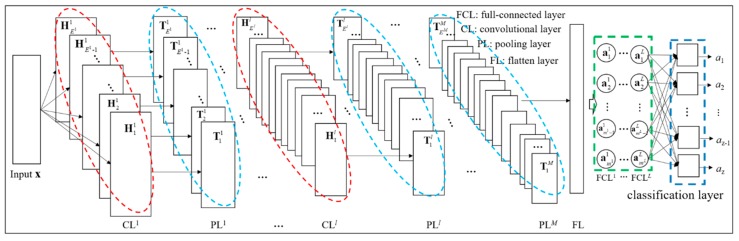
Architecture of a typical convolutional neural network (CNN).

CNN is designed to process data that come in the form of multiple arrays. For example, two-dimensional data for pictures or audio spectrograms and 1D data like signals and sequences [19]. There are four key ideas behind CNN that take advantage of the properties of input data: local connections, shared weights, pooling, and the use of multiple layers [16]. Typically, CNN for classification is composed of five parts [18]: convolutional layers (CL), pooling layers (PL), flatten layer (FL), full-connected layers (FCL), and classification layer, as shown in Figure 2. *M* and *L* represent the total layer number of convolutional layers and full-connected layers respectively. The convolutional and pooling layers are alternated layer by layer for extracting features from input data, the flatten layer is used to transform the outputs of pooling layer into the 1D feature set, and the last two parts are employed for classification from the learned features.

The convolutional layer, as the name suggests, utilizes the operation of convolution to process input data. In one convolutional layer, there are several convolutional kernels (or filters), for example *E^l^* convolutional kernels at the *l*th (l∈[1,2,…,L]) layer. Each convolutional kernel consists of a certain number of trainable weights. One convolutional feature at *l*th layer is calculated as follows:

(2) $$H_{i}^{l}(j)=f(p^{l}(j)*w_{i}^{l}+b_{i}^{l})$$

where the symbol of asterisk * denotes the convolutional operation; $H_{i}^{l}(j)$ represents the convolutional feature of *i*th convolutional kernel that slides to the *j*th region of the input vector, where $i\in [1,2,\ldots ,E^{l}]$; *f* represents the activation function; $p^{l}(j)$ represents the vector composed of the elements in *j*th region of the input vector; $w_{i}^{l}=\left[w_{i,1}^{l},w_{i,2}^{l},\ldots ,w_{i,r^{l}}^{l}\right]$; and $b_{i}^{l}$ denote the *i*th convolutional kernel and its bias. 

The activation function is defined as Rectified Linear Units (ReLU) function, which has more simple derivative result than traditional tanh and sigmoid functions leading to faster training when using the training algorithm. The mathematical expression of the ReLU function is shown as follows:

(3) $$f(x)=\left\{ \begin{array}{l} x,x\geq 0\\ 0,x<0 \end{array}\right.$$

Each convolutional kernel slides over the input vector, then all the convolutional features constitute the convolutional output $H_{i}^{l}$ which is obtained by the *i*th convolutional kernel:

(4) $$H_{i}^{l}=[H_{i}^{l}(1),H_{i}^{l}(2),\ldots ,H_{i}^{l}(n^{l}-r^{l}+1)]$$

Different convolutional kernels attain different convolutional outputs, which fuse information of the input vector from different ways. That is to say, different perspectives of features that are beneficial to classification can be extracted. Furthermore, the convolutional layer provides characteristics of local connections and shared weights, which can reduce the number of parameters, significantly reduce the computational costs, and have certain robustness to local noise [19].

The pooling layer is usually connected after the convolutional layer, which is used to sub-sampling features with maximum pooling, average pooling or other operation. Assuming the pooling size is *c*, the maximum pooling feature of the *j*th region at *l*th pooling layer can be expressed as:

(5) $$T_{i}^{l}(j)=\underset{j}{\max}(H_{i}^{l}(j))$$

where $T_{i}^{l}(j)$ represents the pooling feature when slides to the *j*th region of the *i*th convolutional output and $H_{i}^{l}(j)$ represents the *j*th region of the *i*th convolutional output.

The vector composed of all pooling features is the pooling output denotes as $T_{i}^{l}$ after sliding. The key idea of pooling is to reduce the amount of data transferred to the next layer. Moreover, the pooling takes typical features as its outputs, which owns the ability of invariance [19].

**Figure 3:**
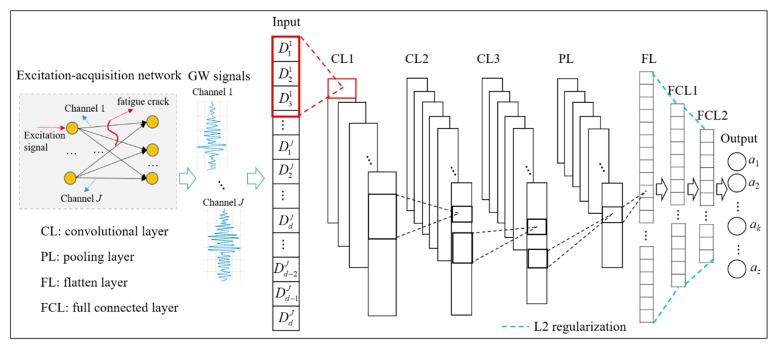
Structure of proposed fatigue crack diagnosis (FCD) CNN model.

In consequence, the framework of the proposed CNN based FCD method can be described in Figure 3. It is noticeable that the proposed CNN model has a nine-layer configuration, which consists of one input layer, three convolutional layers followed with one pooling layer, a flatten layer, two full-connected layers, and an output layer. In the CNN model, to obtain features that are less affected by uncertainties, the first convolutional layer (CL1) is used to extract features from a GW signal and fuse crack information from different channels of GW signals at the same time. As mentioned above, DIs are arranged into an input vector in order of channel. Therefore, the crack information from the same channel is in *d* DIs that are arranged sequentially in the input feature vector. Accordingly, based on the above analysis, a set of kernels with the size of d×1 are utilized in CL1. In this study, each element of the feature vector contains useful crack information and the number of elements is really scarce. However, a pooling layer may lead to the loss of valuable information because it only chooses one typical feature as its output in a pooling area. Hence, there is no pooling layer followed by CL1, but two convolutional layers are added as CL2 and CL3. Taking the Pyramid Shape mentioned in Leslie’s paper [25] into consideration, the kernel size decreases throughout the architecture to get better performance. Next, a pooling layer (PL) is added followed by a flatten layer which is used to transform the outputs of PL into the 1D feature set. Then, two full-connected layers (FCL) are added, denoted by FCL1 and FCL2. The outputs of the flatten layer are employed as the inputs of FCL1. These two full-connected layers are designed as hidden layers to map the features into CNN outputs and select the features that are less affected by uncertainties. Most features that less affected by uncertainties will be found, which are in favor of leading to a higher FCD accuracy. In addition, the L2 regularization method [26] is operated in full-connected layers during the training process to reduce the possibility of overfitting.

The training of CNN is a procedure of optimizing the connected weights and bias, which are trained via minimizing the cost function with training data. In the proposed method, training data are input vectors at different crack length collected from historical data as mentioned above. The cost function of the proposed model is defined as the cross-entropy function in accordance with the softmax classifier which is the output layer of the model, given as the following equation:

(6) $$G(w;q_{k},a_{k})=-\frac{1}{z}\sum_{k=1}^{z}\left[q_{k}lna_{k}+(1-q_{k})\ln(1-a_{k}\right)]+\frac{\lambda }{2}\sum_{l=1}^{L}\sum_{j=1}^{m^{l}}\sum_{i=1}^{m^{l+1}}{\left(w_{j,i}^{l+1}\right)}^{2}$$

where $w_{j,i}^{l}$ denotes the weight that connects the *j*th neuron at the *l*th layer and the *i*th neuron at the (*l* + 1) th layer and *m^l^* denotes the neuron number at *l*th layer. In Equation (6), the first term is the cross-entropy between the CNN output and the desired output, and the second term is the L2 regularization part, in which λ(0<λ<1) denotes the regularization coefficient. Here, weights are initialized with Xavier method [27], which makes the weights of each layer obey the following uniform distribution:

(7) $$w\sim U[-\frac{\sqrt{6}}{\sqrt{m^{l}+m^{l+1}}},\frac{\sqrt{6}}{\sqrt{m^{l}+m^{l+1}}}]$$

Bias is initialized to be *b^l^* = 0. Then, weight and bias are updated with the Adam optimization method [28], in which independent adaptive learning rate is designed for different parameters by calculating the first and second order moment estimation of the gradient. In the Adam optimization method, the first-order moments of weight gradient and bias gradient are initialized to be $V_{dw^{l}}=0,V_{db^{l}}=0$ and the second-order moments are initialized to be $I_{dw^{l}}=0,I_{db^{l}}=0$ firstly. Next, based on the backpropagation algorithm, the recurrence relation for the sensitivity can be written as follows:

(8) $$s_{j}^{l}=(f^{l})\prime \left(x\right){\left(w_{j,i}^{l+1}\right)}^{T}s_{i}^{l+1}$$

where $s_{j}^{l}$ and $s_{i}^{l+1}$ denote the sensitivity of *j*th neuron at the *l*th layer and the *i*th neuron at the (*l* + 1)th layer and $(f^{l})\prime \left(x\right)$ denotes the derivative of the activation function at *l*th layer. Then, the gradient can be calculated by the following expression:

(9) $$dw^{l}=s^{l}a^{l},db^{l}=s^{l}$$

After that, *V*_d*w*_, *V*_d*b*_, *I*_d*w*_*,* and *I*_d*b*_ can be updated as follows:

(10) $$V_{dw^{l}}(k+1)=\beta_{1}V_{dw^{l}}(k)+(1-\beta_{1})dw^{l},V_{db^{l}}(k+1)=\beta_{1}V_{db^{l}}(k)+(1-\beta_{1})db^{l}$$

(11) $$I_{dw^{l}}(k+1)=\beta_{2}I_{dw^{l}}(k)+(1-\beta_{2})(dw^{l}{)}^{2},I_{db^{l}}(k+1)=\beta_{2}I_{db^{l}}(k)+(1-\beta_{2})(db^{l}{)}^{2}$$

where *k* denotes the number of iterations and $\beta_{1}$ and $\beta_{2}$ are parameters that are generally preset as 0.9 and 0.999, respectively. Then, the updated values are corrected as follows:

(12) $$V_{dw^{l}}^{{}^{\mathrm{corrected}}}(k+1)=\frac{V_{dw^{l}}(k+1)}{1-\beta_{1}(k)},V_{db^{l}}^{{}^{\mathrm{corrected}}}(k+1)=\frac{V_{db^{l}}(k+1)}{1-\beta_{1}(k)}$$

(13) $$I_{dw^{l}}^{{}^{\mathrm{corrected}}}(k+1)=\frac{S_{dw^{l}}(k+1)}{1-\beta_{2}(k)},I_{db^{l}}^{{}^{\mathrm{corrected}}}(k+1)=\frac{S_{db^{l}}(k+1)}{1-\beta_{2}(k)}$$

where the superscript represents the corrected value. Finally, the weight and bias are updated as the following equations:

(14) $$w^{l}(k+1)=w^{l}(k)-\alpha \frac{V_{dw^{l}}^{{}^{\mathrm{corrected}}}(k+1)}{\sqrt{I_{dw^{l}}^{{}^{\mathrm{corrected}}}(k+1)}+\varepsilon }$$

(15) $$b^{l}(k+1)=b^{l}(k)-\alpha \frac{V_{db^{l}}^{{}^{\mathrm{corrected}}}(k+1)}{\sqrt{I_{db^{l}}^{{}^{\mathrm{corrected}}}(k+1)}+\varepsilon }$$

where α denotes the learning rate and ε is set to prevent the denominator from being 0, which can be $\varepsilon =10^{-8}$.

The training of CNN is finished when the cost function is close to converging. Eventually, the input feature vector from a similar monitored structure is put into the trained model to get its diagnostic crack size.

## 3. Experimental Verification and Analysis

Attachment lugs are an important type of joint in aircraft structures. They are typically used as a connection between two components, and are quite susceptible to fatigue cracks due to stress concentrations. In this paper, fatigue tests are performed on attachment lug specimens to validate the proposed method. The uncertainty of GW signals and DIs are discussed. After that, the designed CNN model and its training process are given. Finally, the fatigue crack diagnosis results based on the proposed method are presented and discussed.

### 3.1. Fatigue Tests of Attachment Lug Specimens

**Figure 4:**
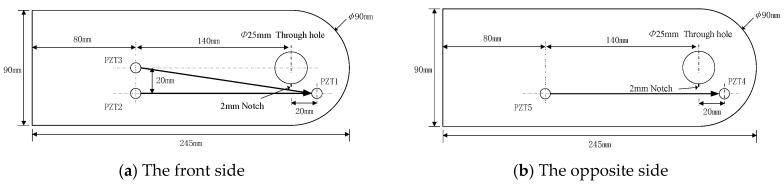
Geometry and sensor layout of the attachment lug.

**Figure 5:**
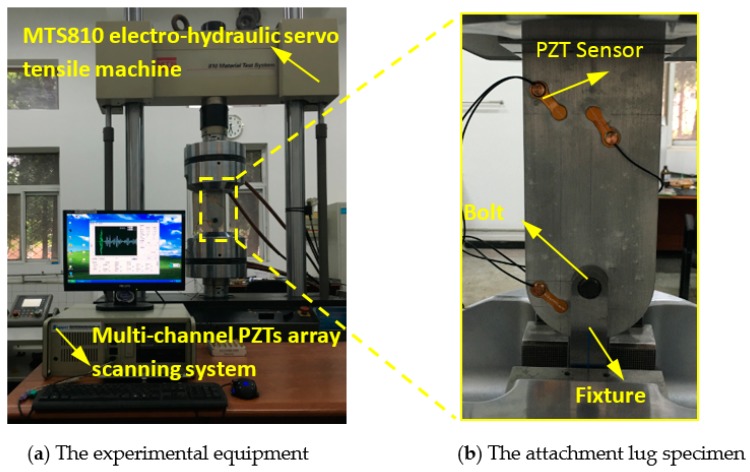
The fatigue test setup.

Fatigue tests are performed on six attachment lug specimens, labeled from T1 to T6. To be close to the real engineering applications, the specimens are made of 5 mm-thick LY12 aluminum alloy, which have a hole with the diameter of 25 mm. Their geometry and sensor layout are shown in Figure 4. In real engineering applications, the attachment lug usually suffers from the axial tension load during service. Finite element method results for the lug show that stress concentration occurs at the edge of the hole. Since the stress concentration area often initiates crack, a 2 mm long notch is made at the edge of the lug hole to make the crack initiation and control the direction of crack growth, and this notch was created by a wire-electrode cutting machine. The same sensor layout is chosen for each specimen, in which PZT sensors named PZT1–PZT3 are arranged on the front side and PZT4–PZT5 are arranged on the opposite side. As shown in Figure 5b, a fixture with a dowel pin is adopted to connect the lug, and transmits the axial tension load, which is a real load transmitting style in engineering applications.

**Figure 6:**
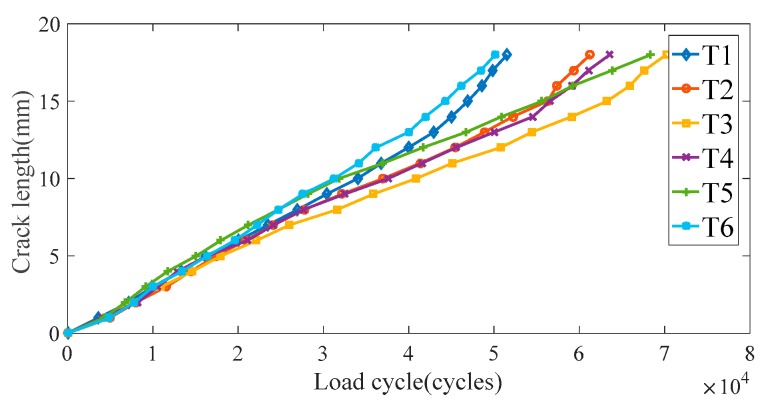
Crack growth of specimens T1–T6.

An MTS810 electro-hydraulic servo tensile machine is used to apply the fatigue load, as shown in Figure 5a. According to engineering experience, a sinusoidal load with a peak value of *F*_max_ = 18 kN is chosen for fatigue tests, which is 25% of the fracture load of 72kN. The load frequency is chosen as 10Hz and the stress ratio is R = *F*_min_/*F*_max_ = 0.1. The crack lengths were measured with a digital magnifier and scale lines on one surface of the specimen. The crack growth processes of specimens T1*–*T6 are shown in Figure 6. It is noteworthy that crack growth of different specimens is different; this will introduce uncertainties for FCD.

During the fatigue tests, the multi-channel PZTs array scanning system developed by the authors’ group is employed to perform the GW based fatigue crack monitoring [29]. Here, the three-cycle sine burst signal with central frequency of 160 kHz and the exciting voltage of ±70 V is adopted as exciting signal, and the sampling frequency is set as 50MHz. Moreover, PZT2, PZT3, and PZT5 are chosen to excite signals, while PZT1 and PZT4 are used to acquire signals. As a result, three effective exciting-acquisition channels are obtained denoted as 2-1 channel, 3-1 channel, and 5-4 channel as shown in Figure 4. The fatigue load is suspended to collect two sets of GW signals when the fatigue crack grows every 1 mm for the crack length of 0 mm to 18 mm, where each set contains three channels of GW signals.

**Figure 7:**
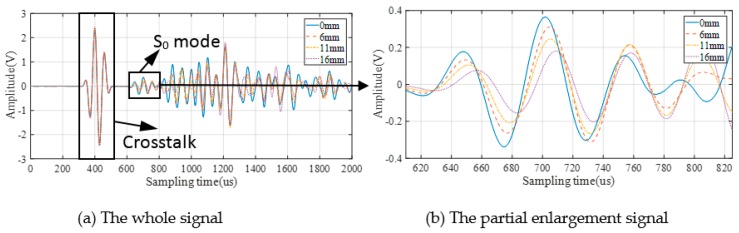
GW signals of 2-1 channel in specimen T1.

**Figure 8:**
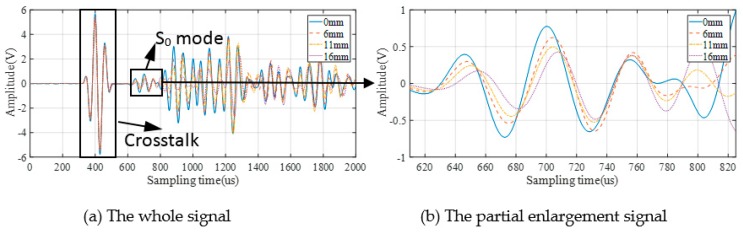
GW signals of 2-1 channel in specimen T4.

As a consequence, 38 sets of signals are collected from each specimen, and 228 set of signals for six specimens in total. Figure 7 and Figure 8 show the GW signals of specimens T1 and T4 under partial crack length during the fatigue crack monitoring in the 2-1 channel.

It can be seen from the above figures that GW signals obtained from different specimens (T1/T4) are different in both amplitude and phase. These are the reflection of uncertainties among different structures on original GW signals.

### 3.2. GW Monitoring and DIs Extraction

According to Figure 7 and Figure 8, the S_0_ mode which has a clear wave packet is chosen to calculate DIs. However, the other part of the signal is affected by complex mode, boundary reflection, and so on, which makes it difficult to extract the crack information from the mix of these waves. In addition, for different specimens, the difference of signal behind S_0_ in different specimens is much greater than that of S_0_ mode in different specimens. That will make the crack quantification problem more difficult. Seven kinds of DIs are used for GW features extraction as shown in Table 1, where *B*(*t*) and *D*(*t*) are the baseline signal and the monitoring signal, respectively; the baseline is the GW signal acquired after the notch is machined and it is unique to each sample; *t*_1_ and *t*_2_ are the start time and the end time of the selected wave packet; and ω denotes the signal frequency obtained by the Fourier transform. 

As is shown in the table, These DIs reflect the crack from the time domain and frequency domain, which can be more comprehensive response to crack length.

1. The Cross correlation is a DI in the time domain, which is describe the degree of correlation between the normalized baseline signal and the monitoring signal;
2. The Spatial phase difference is also a DI in the time domain that describes the size of the angle between the normalized baseline signal and the monitoring signal;
3. The Spectrum loss is a DI in the frequency domain, which describes the difference value in spectrum between two signals;
4. The Central spectrum loss is also a DI in the frequency domain, which measures the change of central spectrum between baseline and monitoring signal;
5. The Differential curve energy is a DI that measured the variation of waveform curve of the difference of two signal;
6. The Normalized Correlation Moment is a DI that based on local statistical features of the waveform; the energy and phase change of the signal has been taken into consideration;
7. The differential signal energy is a DI that measured the variation of signal energy.

**Figure 9:**
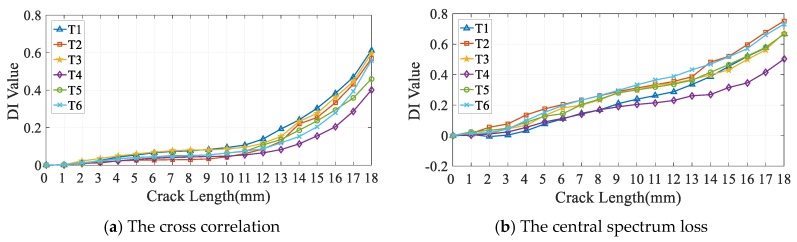
DI of 2-1 channel in specimens T1–T6.

To give an intuition of what the uncertainty of DIs is and show how the multi-channel DIs fusing owns the potential of improving the accuracy of FCD. Firstly, typical time-domain DI (cross correlation) and frequency-domain DI (central spectrum loss) varying with crack growth in the same typical 2-1 channel are shown in Figure 9.

It is apparent that these DIs can reflect the fatigue crack growth effectively, but there is a large uncertainty of the same DI values under the same fatigue crack length for different specimens, which means the same DI value corresponds to multiple crack lengths. Meanwhile, numerical distributions of different DIs are altered during the fatigue crack growth process for the same specimen, which indicates that the influence of crack on GW signal is described from different perspectives by different DIs. This provides a basis for data fusion with CNN.

**Figure 10:**
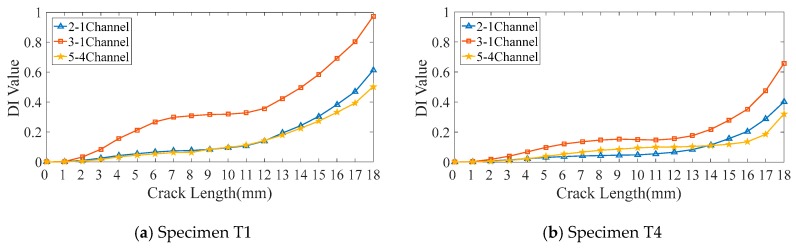
Different channels of cross-correlation DI.

Then, the cross-correlation DI is chosen to show its value varying with fatigue crack growth for typical specimen T1 and T4 under three different exciting-acquisition channels. As shown in Figure 10, the distribution of DIs in different exciting-acquisition channels are different during crack growth, which indicates that different channels of DIs provide different perspectives of crack information for CNN to learn.

As is shown from the above analysis, three channels of GW signals can be acquired under one crack length during the fatigue tests. As for each GW signal, 7 kinds of DIs are extracted. Finally, 7×3×1=21 DIs are obtained under one crack length to construct a 1D feature vector **p**.

### 3.3. GW-CNN Based Diagnosis Training

In this study, all input vectors p that correspond to different crack length are standardized to $p^{norm}$ firstly. Then, the fatigue cracks are classified into 19 crack sizes according to its crack length: 1 crack size per 1mm, named C1–C19, as shown in Table 2. Crack size C*_k_* (k∈[1,2,…,19]) is transformed into the desired output form of q=[0,0,…,1,…,0] in which the *k*th element is 1 and the rest are 0. The standardized input $p^{norm}$ and its corresponding desired output q are formed to input-output pair: $\left\{p_{21\times 1}^{norm},q_{19\times 1}\right\}$ to be an input sample.

In real engineering applications, the fatigue crack information obtained from existing structures is often used to diagnose fatigue crack of unknown structures. Thus, samples from one of the specimens among T1–T6 are chosen as the testing samples, and the rest samples are used for training, as shown in Table 3. Consequently, as for each testing specimen, 38 and 190 samples are utilized for testing and training.

**Figure 11:**
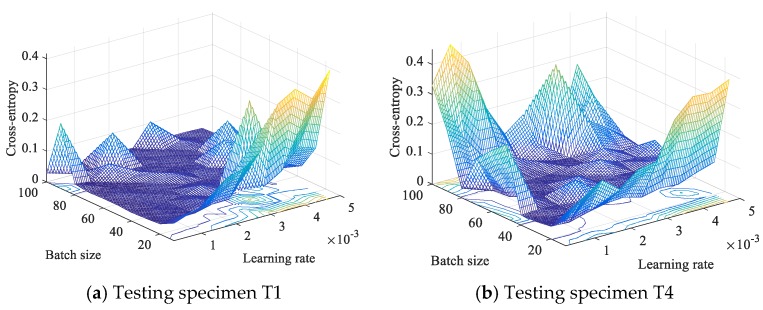
Cross-entropy of the CNN model with different batch size and learning rates.

The algorithm implementation of the designed CNN is under Python on a desktop with Nvidia graphics processing unit (GPU) GTX960. Before the model training, several important hyper-parameters, including learning rate, mini-batch size, kernel size and kernel numbers in convolutional layers and pooling layer, neuron numbers of full-connected layers, and regularization coefficient λ, needed to be determined in advance. In this work, the CNN configuration was set to CL1(16)-CL2(32)-CL3(32)-PL(32)-FCL1(1024)-FCL2(512), and the kernel size was set to CL1(7)-CL2(5)-CL3(5) according to previous studies. The L2 regularization was added to FCL1 and FCL2, and the regularization coefficients were set to be 0.01 and 0.05, respectively. The optimal learning rate and batch size were obtained via a trial-and-error method. Here, various batch sizes were considered, ranging from 10 to 100 with an interval of 10, while the learning rate varies from 0.0002 to 0.005 with an interval of 0.0005. Figure 11 shows the cross-entropy of CNN model for testing specimens T1 and T4. It is noticeable that small values of batch size and large values of learning rate will lead to poor performance (big costs value) of the trained model. With the increase of batch size and decrease of the learning rate, the cross-entropy error of the CNN model will be gradually decreased and then tends to be stable or will be increased a little bit after the costs arrive at its minimum value. According to the result in Figure 11 and a more subtle adjustment, 24 and 0.001 were adopted as the optimal batch size and learning rate for CNN training.

**Figure 12:**
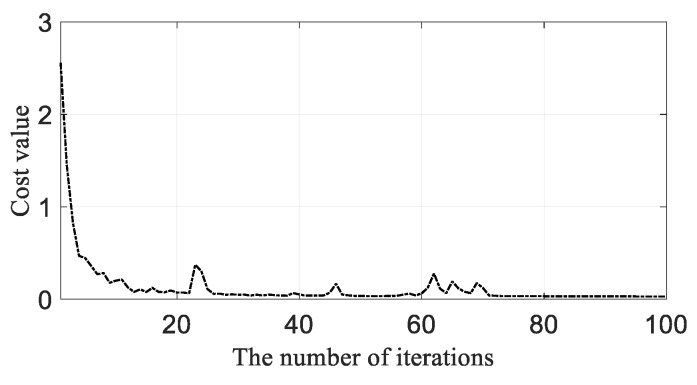
Costs value varying with iteration when the testing specimen is T1.

Then, the CNN model was trained based on optimal model parameters using training samples. In the situation where specimen T1 was used as the testing specimen, the training samples from T2–T6 were put into CNN model to train the model with Adam algorithm. As shown in Figure 12, the CNN model at the iteration of 22 was chosen as the trained CNN model because the costs started to converge and too much training may lead to overfitting. When another specimen was selected as the testing specimen, the corresponding CNN model was trained in the same way above.

### 3.4. Diagnosis Results and Discussion

**Figure 13:**
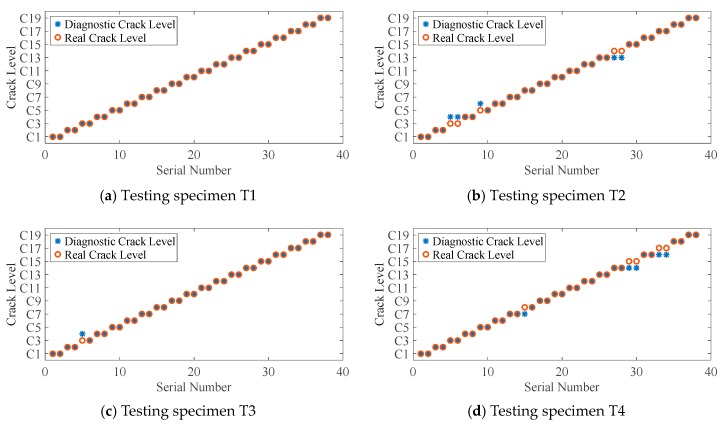

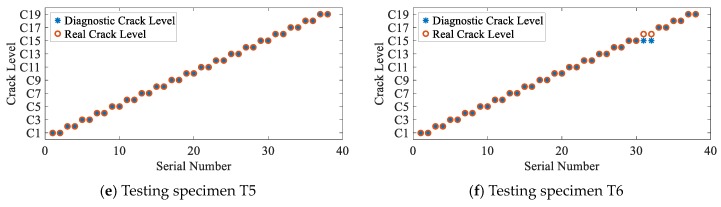
Diagnostic results of different testing specimen.

Testing samples from the testing specimen are put into the trained CNN model to obtain diagnostic crack size. After that, the testing accuracy shown as Table 4 can be calculated as the following equation:

(16) $$\mathrm{Testing}\;\mathrm{accuracy}=\frac{NCC}{NT}\times 100\%$$

where *NCC* denotes the number of correctly classified crack size by CNN and *NT* denotes the total number of testing samples. The CNN diagnostic crack size and its real crack size are given in Figure 13.

**Figure 14:**
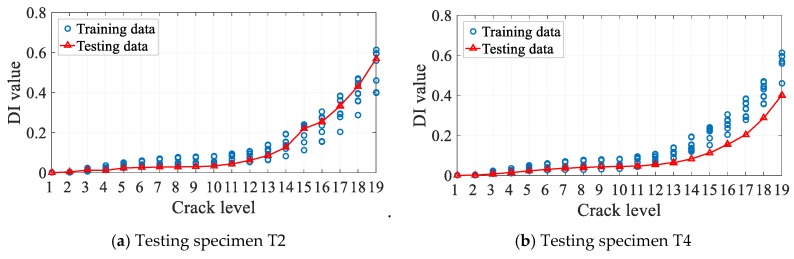
The distribution of cross correlation DI for testing and training data.

Seen from the above results, the diagnostic accuracies of testing specimens T1 and T5 are 100%; as for testing specimens T2 and T4, the accuracies are 86.84%. To display what happened in T2 and T4, the distributions of typical DI (cross-correlation) in the testing data (red triangle) and training data (blue circle) of the 2-1 channel varying with crack sizes are shown in Figure 14, respectively. In Figure 14a, DI values of testing specimen T2 are basically smaller than those of training DI values before C14, but the values become larger after C15. In Figure 14b, DI values of the testing specimen of T4 are basically smaller than DI values of training specimens. These phenomena also happen in other kinds of DIs or other channels, which make it difficult to estimate the fatigue crack size by the general rule of the DI values varying with crack sizes of training data. However, the diagnostic error is only 1 mm as shown in Figure 14, which is allowed in real engineering applications. This indicates that the proposed method is promising in the presence of uncertainties.

To illustrate the effectiveness of the proposed method in solving the problem of uncertainties, the Unified Euclidean Distance is adopted to quantitatively analyze the similarity of input feature vector and features extracted by CNN between two different specimens. Assuming there are two kinds of variables $X=[x_{1},x_{2},\ldots ,x_{i},\ldots ]$ and $Y=[y_{1},y_{2},\ldots ,y_{j},\ldots ]$, in which **x***_i_* = [*x_i_*_1_, *x_i_*_2,…,_ *x_in_*] and $y_{j}=[y_{j1},y_{j2},\ldots ,y_{jm}]$. These two variables are different in variable length (*n* ≠ *m*) and numerical distribution. Taking the Unified Euclidean Distance between specimen 1 and specimen 2 as an example, the Unified Euclidean Distance of $D_{12}^{X}$ between $x_{1}=[x_{11},x_{12},\ldots ,x_{1n}]$ and $x_{2}=[x_{21},x_{22},\ldots ,x_{2n}]$ was calculated as follows: firstly, **x**_1_ and **x**_2_ were normalized to reduce the influence of different numerical distributions expressed as Equation (17); then, the results were divided by the length of the variable *n* to avoid the influence of length as expressed in Equation (18). The Unified Euclidean Distance $D_{12}^{Y}$ between $y_{1}=[y_{11},y_{12},\ldots ,y_{1m}]$ and $y_{2}=[y_{21},y_{22},\ldots ,y_{2m}]$ was calculated in the same way as $D_{12}^{X}$. After that, $D_{12}^{X}$ and $D_{12}^{Y}$ can be used to compare which is more similar between two specimens. The larger the Unified Euclidean Distance is, the less similar the data will be.

(17) $$x\prime_{1i}=x_{1i}/\max(x_{1}),x\prime_{2i}=x_{2i}/\max(x_{2}),i\in [1,2,3,\ldots n]$$

(18) $$D_{12}^{X}=\sqrt{\sum_{k=1}^{n}{\left(x\prime_{1k}-x\prime_{2k}\right)}^{2}}/n$$

Suppose that T1 is the testing specimen, the typical crack sizes C7 and C18 are selected to compare two kinds of Unified Euclidean Distance (a. input feature vector, b. output features from the FCL2, denoted as the output feature vector) between two specimens. Results are shown in Table 5, where $D^{\mathrm{in}}$ and $D^{\mathrm{out}}$ denotes the Unified Euclidean Distance of the input feature vector and output feature vector respectively.

In Table 5, it is apparent that, under the same crack size, Unified Euclidean Distance of output features are smaller than Unified Euclidean Distance of input features between the identical specimens. This shows that output features between training specimens (T2 and T4) or between testing specimen and training specimen (T2 and T1, T4 and T1) are more similar than input features under the same crack size. That is to say, after features fusion and extraction by CNN, the invariance of different specimens under the same crack size is found, which reduces the uncertainties and effectively improves the reliability of FCD. 

Furthermore, two sets of comparative experiments (**I** and **II**) were carried out to verify the advantages of the proposed method for reliable FCD under the influence of uncertainties.

**(I)**. To verify advantages of multi-channel DIs extraction, DIs extracted from a single-channel (2-1 channel, 3-1 channel, and 5-4 channel) were chosen to form the input sample, which has the dimension of 7×1 to be the comparison modes. Notably, the form of the CNN input is the only difference in this comparative experiment.

**Figure 15:**
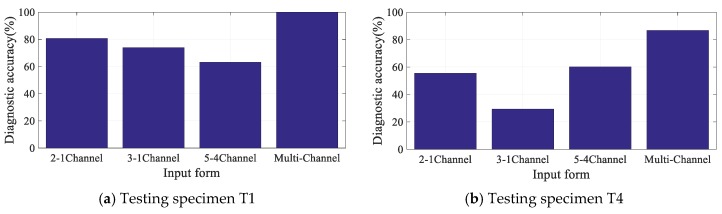
Diagnostic accuracies for typical specimens with different input forms.

Diagnostic accuracies of specimens T1 and T4 for three single-channel input forms and multi-channel input form are shown in Figure 15, respectively. Seen from the figures, diagnostic accuracies for single-channel input forms are lower than 80% for T1 and lower than 60% for T4. This indicates that the uncertainty of DIs seriously affects the diagnostic accuracies. Compared with the single-channel input forms, the diagnostic accuracies for multi-channel input form are 100% and 86.84% for T1 and T4. The multi-channel input vector contains more crack information for the designed CNN to extract features that are beneficial to classification, leading to the improvement of the diagnostic accuracy effectively.

**(II)**. Traditional neural networks (backpropagation networks) and the softmax classifier are selected as comparative classification methods to show the advantages of designed CNN model. For one type, the neural number of single hidden layer are set which has the same neural number (NN(1024)) and different neural number (NN(100)) as FCL1, respectively. For another type, the neural number of multiple hidden layer are set which has the same neural number (NN(1024-512)) and different neural number (NN(512-100)) as FCL1 and FCL2, respectively. The purpose of these settings is to compare the effectiveness of convolutional layers. The neural number of the softmax classifier is 19. It is worth noting that different classification methods are trained and tested with the same training and testing input samples.

**Figure 16:**
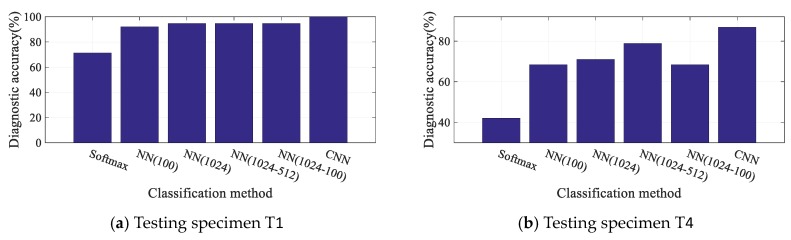
Diagnostic accuracies for typical specimens with different classification methods.

The diagnostic accuracies and training time of testing specimens T1 and T4 are shown in Figure 16 and Table 6, respectively. In Figure 16, the proposed method achieves the highest diagnostic accuracy in both T1 and T4. That is to say, the reliable FCD is difficult to be realized only by full-connected neural networks because of the lack of expression ability. Table 6 shows that the training consumption of the proposed method has been dramatically reduced compared with traditional methods indicating high diagnostic efficiency of the proposed method.

## 4. Conclusions

This article puts forward a GW-CNN based approach to perform FCD under the influence of uncertainties due to different structures in real engineering applications. Firstly, multi-channel DIs under one crack length were extracted to construct an input feature vector to obtain more crack information for CNN to learn. Then, a CNN was designed to fuse multi-perspective fatigue crack information from the feature vector to realize reliable FCD. Moreover, data standardization, L2 regularization, and an Adam algorithm were adopted to improve the performance of CNN. Finally, CNN was trained with input vectors at different crack length from historical data. What is more, the proposed method was verified with attachment lug specimens. The lowest diagnostic accuracy was 86.84%, which has a diagnostic error of only 1 mm. Results show that the proposed method effectively reduced the influence of uncertainties on FCD between different structures. Finally, two sets of comparative experiments were carried out. Compared with the single-channel input sample form, the proposed method obtained much better diagnostic robustness and accuracy. Compared with the traditional methods, the designed CNN not only improves the accuracy of FCD, but also reduces the training consumption. There are some points for ongoing work to take verification on real structures or in operating conditions, considering the time-varying temperature and loading conditions.

## Figures and Tables

**Table 1:** DI extraction algorithms.

Damage IndexDI	Extraction Algorithm
Cross correlation [6]	$DI=1-\sqrt{\frac{{\left[\int_{t_{1}}^{t_{2}}B\left(t\right)D\left(t\right)dt\right]}^{2}}{\int_{t_{1}}^{t_{2}}B^{2}\left(t\right)dt\int_{t_{1}}^{t_{2}}D^{2}\left(t\right)dt}}$
Spatial phase difference	$DI=\int_{t_{1}}^{t_{2}}{\left(\tilde{D}(t)-\alpha B(t)\right)}^{2}dt,\tilde{D}(t)=\frac{D\left(t\right)}{\sqrt{\int_{t_{1}}^{t_{2}}D^{2}(t)dt}},\alpha =\frac{\int_{t_{1}}^{t_{2}}\tilde{D}(t)B(t)dt,}{\int_{t_{1}}^{t_{2}}B^{2}(t)dt}$
Spectrum loss	$DI=\frac{\int_{\omega_{1}}^{\omega_{N}}\left|B\left(\omega \right)-D\left(\omega \right)\right|d\omega }{\int_{\omega_{1}}^{\omega_{N}}\left|B\left(\omega \right)\right|d\omega \;}$
Central spectrum loss	$DI=\frac{a\left(\omega \right)-b\left(\omega \right)}{a\left(\omega \right)\;},a\left(\omega \right)=\max(B\left(\omega \right)),b\left(\omega \right)=\max(D\left(\omega \right))$
Differential curve energy [7]	$DI=\frac{\sum_{n=2}^{N}{\left[b(t_{n})-b(t_{n-1})\right]}^{2}}{\sum_{n=2}^{N}{\left[B(t_{n})-B(t_{n-1})\right]}^{2}},b(n)=B(n)-D(n)$
Normalized Correlation Moment [8]	$DI=\frac{\int_{\tau =t_{1}}^{\tau =t_{2}}\tau^{k}\left|r_{HH}(\tau )\right|d\tau -\int_{\tau =t_{1}}^{\tau =t_{2}}\tau^{k}\left|r_{HD}(\tau )\right|d\tau }{\int_{\tau =t_{1}}^{\tau =t_{2}}\tau^{k}\left|r_{HH}(\tau )\right|d\tau },r_{HD}(\tau )=\int_{-\infty }^{+\infty }B(t)D(t-\tau )dt,r_{HH}(\tau )=\int_{-\infty }^{+\infty }B(t)B(t-\tau )dt$
Differential signal energy	$DI=\int_{t_{1}}^{t_{2}}{\left(\tilde{B}\left(t\right)-\tilde{D}\left(t\right)\right)}^{2}dt,\tilde{B}\left(t\right)=\frac{B\left(t\right)}{\sqrt{\int_{t_{1}}^{t_{2}}B^{2}\left(t\right)dt}},\tilde{D}\left(t\right)=\frac{D\left(t\right)}{\sqrt{\int_{t_{1}}^{t_{2}}D^{2}\left(t\right)dt}}$

**Table 2:** Fatigue crack sizes corresponding to crack length.

**Crack Size**	**C1**	**C2**	**C3**	**C4**	**C5**	**C6**	**C7**	**C8**	**C9**	**C10**
Crack length (mm)	0	1	2	3	4	5	6	7	8	9
**Crack size**	**C11**	**C12**	**C13**	**C14**	**C15**	**C16**	**C17**	**C18**	**C19**	
Crack length (mm)	10	11	12	13	14	15	16	17	18	

**Table 3:** Testing specimen and its corresponding training specimens.

Testing Specimen	T1	T2	T3	T4	T5	T6
Training specimens	T2–T6	T1, T3–T6	T1–T2, T4–T6	T1–T3, T5–T6	T1–T4, T6	T1–T5

**Table 4:** Diagnostic results of different testing specimen.

Testing Specimen	T1	T2	T3	T4	T5	T6
Accuracy	100%	86.84%	97.37%	86.84%	100%	94.74%

**Table 5:** Unified Euclidean Distance between different specimens.

Crack Size	Input Samples			Output Features		
	$D_{T2,T4}^{\mathrm{in}}$	$D_{T2,T1}^{\mathrm{in}}$	$D_{T4,T1}^{\mathrm{in}}$	$D_{T2,T4}^{\mathrm{out}}$	$D_{T2,T1}^{\mathrm{out}}$	$D_{T4,T1}^{\mathrm{out}}$
C7	2.0159	0.2142	1.8326	0.0002	0.0016	0.0011
C18	0.0204	0.0253	0.0137	0.0002	0.0011	0.0015

**Table 6:** Training time for typical specimens with different classification methods.

Classification Method	Softmax	NN (100)	NN (1024)	NN (1024-512)	NN (512-100)	CNN
T1	89 s	59 s	33 s	89 s	50 s	14 s
T4	35 s	117 s	117 s	86 s	57 s	9 s
